# Novel ACE-Inhibitory Peptides from Royal Jelly Proteins: Comprehensive Screening, Mechanistic Insights, and Endothelial Protection

**DOI:** 10.3390/foods15010084

**Published:** 2025-12-26

**Authors:** Wanyu Yang, Xinyu Zou, Tianrong Zhang, Qingqing Liu, Ziyan Liu, Fan Li, Yuhong Luo, Yiwen Wang, Zhijun Qiu, Bin Zhang

**Affiliations:** 1 College of Food and Bioengineering, Henan University of Science and Technology, Luoyang 471023, China; 230320070641@stu.haust.edu.cn (W.Y.); zouxinyu@stu.haust.edu.cn (X.Z.); zhangtianrong1002@163.com (T.Z.); lqq11276245@outlook.com (Q.L.); zyliu1004@163.com (Z.L.); lifannn1217@163.com (F.L.); luoyh0521@163.com (Y.L.); 251416120424@stu.haust.edu.cn (Y.W.); qiuzj2003@163.com (Z.Q.); 2 Henan Engineering Research Center of Food Microbiology, Luoyang 471023, China; 3 National Demonstration Center for Experimental Food Processing and Safety Education, Luoyang 471023, China

**Keywords:** ACE-inhibitory peptide, royal jelly, virtual screening, molecular docking, EA.hy926 cell

## Abstract

This study aimed to identify novel angiotensin-converting enzyme (ACE)-inhibitory peptides from royal jelly proteins (RJPs) by integrating *in silico* digestion, virtual screening, and *in vitro* evaluation. Three major royal jelly proteins (MRJP1-3) were subjected to *in silico* digestion using 16 enzymatic systems, yielding 1411 unique peptides. Virtual screening based on predicted bioactivity, toxicity, water solubility, and ADMET profiles resulted in the selection of 27 candidate peptides. Molecular docking revealed strong binding affinities for these peptides compared with the positive control captopril, among which PYPDWSFAK and RPYPDWSF exhibited potent ACE-inhibitory activity, with IC_50_ values of 110 ± 1.02 μmol/L and 204 ± 0.61 μmol/L, respectively. Kinetic analysis indicated that PYPDWSFAK acts as a mixed-type ACE inhibitor. Docking visualization demonstrated that PYPDWSFAK forms multiple hydrogen bonds with key residues in the ACE active pocket and directly coordinates with the catalytic Zn^2+^ ion. Cellular assays showed that PYPDWSFAK was non-cytotoxic, suppressed Ang II–induced endothelial cell migration, restored NO and ET-1 balance, and enhanced SOD and GSH-Px activities. Overall, this study enriches the repertoire of ACE-inhibitory peptides derived from royal jelly proteins. Furthermore, PYPDWSFAK is identified as a promising ACE-inhibitory peptide with potential for incorporation into natural antihypertensive ingredients or functional foods.

## 1. Introduction

Hypertension is a prevalent chronic condition and a major contributor to cardiovascular morbidity and mortality worldwide [1]. Persistent hypertension can induce oxidative stress and endothelial dysfunction, accelerating vascular injury and further emphasizing the need for safer, long-term therapeutic strategies [2]. As one of the key therapeutic targets for hypertension management, angiotensin-I-converting enzyme (ACE, EC 3.4.15.1) plays a pivotal role in blood pressure regulation through the renin-angiotensin system [3]. Although synthetic ACE inhibitors, such as captopril, enalapril, and lisinopril, are effective in hypertension management, their long-term use is frequently associated with adverse side effects, including dry cough, angioedema, hyperkalemia, and hypotension [4]. Compared to synthetic drugs, food-derived bioactive peptides are generally considered safer, with advantages such as low toxicity, good biocompatibility, high gastrointestinal absorption, and minimal side effects [5]. In recent years, numerous ACE-inhibitory peptides have been identified from a variety of dietary sources, such as soy protein [6], *Mytilus edulis* [7], and whey protein [8]. These studies have demonstrated that food-derived ACE-inhibitory peptides can serve as effective and natural alternatives to the synthetic inhibitors in hypertension management [9].

As compared to traditional experiment-based screening approaches for ACE-inhibitory peptides, which are time-consuming and labor-intensive [10], an *in silico* strategy that integrates peptidomic analysis with virtual screening has emerged as a more efficient and cost-effective approach for identifying ACE-inhibitory peptides from food resources [11]. For example, in a previous study by our group, a novel ACE-inhibitory peptide, MMDF, was identified from the simulated digest of oyster proteins using a virtual screening pipeline that incorporated bioactivity prediction, physicochemical property evaluation, ADMET analysis, and molecular docking [12]. In addition, by integrating LC-MS/MS technology with virtual screening and molecular docking analysis, a novel ACE-inhibitory peptide FPPDVA was identified from tuna dark muscle hydrolysates, and its inhibitory activity was further confirmed through an *in vitro* ACE assay with an IC_50_ value of 87.11 ± 1.02 μmol/L [13]. Furthermore, many additional ACE-inhibitory peptides have been identified through similar computational-assisted workflows from various protein sources, including rabbit meat protein [14], garlic protein [10], and rapeseed protein [15]. While *in silico* prediction provides valuable guidance for identifying potential peptide candidates, experimental validation is essential to confirm their biological relevance. Furthermore, *in vivo* investigations and long-term studies are necessary to comprehensively evaluate their efficacy and safety as food supplements or functional nutrients.

In recent years, the hydrolysates of royal jelly proteins (RJPs) have gained increasing attention due to their broad spectrum of biological activities, including antioxidant [16], antibacterial [17], anti-aging [18], and antihypertensive properties [19], which are largely attributed to their favorable amino acid composition and sequence characteristics. In a previous study, both the pepsin hydrolysate of RJPs (IC_50_ = 0.358 mg protein/mL) and the hydrolysate generated by the combination of pepsin, trypsin, and chymotrypsin (IC_50_ = 0.099 mg protein/mL) exhibited strong *in vitro* ACE-inhibitory activity and significantly reduced systolic blood pressure in spontaneously hypertensive rats (SHRs) [20]. In addition, three ACE-inhibitory peptides, including IY, VY, and IVY, have been identified from the RJP hydrolysate prepared using Protease N, and these peptides showed notable antihypertensive effects following repeated oral administration for 28 days in SHRs [21]. In addition, in our previous research, we found that all 49 peptides identified from the simulated digest of RJPs were predicted to possess ACE-inhibitory potential using the BIOPEP-UWM database (unpublished data). Taken together, these findings suggest that RJPs represent an abundant and promising source for the discovery of potent ACE-inhibitory peptides. However, peptidomics-based characterization of RJP hydrolysates is still lacking, and only a limited number of ACE-inhibitory peptides have been reported so far, indicating that the ACE-inhibitory potential of RJPs remains far from fully explored.

In this study, with a particular focus on the efficiency of the *in silico* approach, we systematically screened RJPs for potential ACE-inhibitory peptides by an integrated workflow encompassing *in silico* digestion, virtual screening, and molecular docking. Based on these *in silico* predictions, the most promising candidates were synthesized, and their inhibitory activity and kinetic characteristics were subsequently verified through *in vitro* ACE assays. The mechanism and cellular protective effects of the lead peptide were further examined using EA.hy926 endothelial cells by assessing migration, NO and ET-1 balance, and antioxidant enzyme activities. Collectively, this integrated strategy establishes a robust and efficient workflow for functional peptide discovery and reveals new ACE-inhibitory peptides from RJPs with strong antihypertensive potential.

## 2. Materials and Methods

### 2.1. Materials and Reagents

Angiotensin-converting enzyme (ACE) was purchased from Sigma-Aldrich (St. Louis, MO, USA). Hippuryl-histidyl-leucine (HHL) was obtained from Aladdin Biochemical Technology Co., Ltd. (Shanghai, China). EA.hy926 cells, High-glucose DMEM, fetal bovine serum (FBS), and penicillin-streptomycin (P/S) were obtained from Wuhan Procell Life Science & Technology Co., Ltd. (Wuhan, China). Trypsin-EDTA was purchased from Beijing Solarbio Science & Technology Co., Ltd. (Beijing, China). Angiotensin II (Ang II), nitric oxide (NO) assay kit, and endothelin-1 (ET-1) assay kit were purchased from Sangon Biotech Co., Ltd. (Shanghai, China). The BCA protein assay kit, glutathione peroxidase (GSH-Px) assay kit, and superoxide dismutase (SOD) assay kit were obtained from Nanjing Jiancheng Bioengineering Institute (Nanjing, China).

All other chemicals and reagents used in the study were of analytical grade unless otherwise specified.

### 2.2. In Silico Hydrolysis of RJPs

Royal jelly is rich in major royal jelly proteins, which account for more than 80% of its total protein content, mainly comprising MRJP1, MRJP2, and MRJP3 [22,23]. Given their abundance and reported bioactivities, these three proteins were selected as representative substrates for *in silico* hydrolysis. The amino acid sequences of MRJP1 (O18330), MRJP2 (O77061), and MRJP3 (Q17060) were retrieved from the UniProt database (https://www.uniprot.org (accessed on 20 June 2024)).

To generate a peptide sequence pool for subsequent virtual screening, 15 proteases were selected, including 10 animal proteases, i.e., pepsin (EC 3.4.23.1), trypsin (EC 3.4.21.4), chymotrypsin (A) (EC 3.4.21.1), chymotrypsin C (EC 3.4.21.2), metridin (EC 3.4.21.3), pancreatic elastase (EC 3.4.21.36), leukocyte elastase (EC 3.4.21.37), cathepsin G (EC 3.4.21.20), chymase (EC 3.4.21.39), and plasmin (EC 3.4.21.7); 3 plant proteases, i.e., papain (EC 3.4.22.2), stem bromelain (EC 3.4.22.32), and ficin (EC 3.4.22.3); and 2 microbial proteases, i.e., thermolysin (EC 3.4.24.27) and proteinase K (EC 3.4.21.64). In addition, a combined digestion using pepsin and pancreatin was also employed to simulate gastrointestinal hydrolysis of RJPs. The *in silico* hydrolysis was conducted using the “Enzyme(s) action” tool in the BIOPEP-UWM database (https://biochemia.uwm.edu.pl/biopep/rec_pro4.php (accessed on 20 June 2024)).

### 2.3. Virtual Screening of the Peptides from the in Silico Digests of RJPs

The RJP-derived peptides were virtually screened based on their predicted bioactivity, toxicity, water solubility, and human intestinal absorption (HIA), with the aim of identifying ACE-inhibitory candidates suitable for functional food and healthcare applications. The predicted bioactivities of the peptides were evaluated using the PeptideRanker tool (http://distilldeep.ucd.ie/PeptideRanker/ (accessed on 10 July 2024)), with peptides exhibiting scores > 0.8 considered bioactive candidates [24]. The water solubility of the selected peptides was further assessed using the Peptide Property Calculator (http://www.innovagen.com/proteomics-tools (accessed on 11 July 2024)). In addition, key physicochemical parameters, such as molecular weight, isoelectric point (pI), and net charge at physiological pH 7.0, were also determined to support peptide selection. The toxicity of the selected peptides was predicted using the ToxinPred server (http://webs.iiitd.edu.in/raghava/toxinpred/ (accessed on 15 July 2024)) [25]. The ADMET profiles, including HIA, blood–brain barrier (BBB) penetration, and cytochrome P450 (CYP450) 2D6 interaction, were analyzed using the admetSAR 2.0 platform (http://lmmd.ecust.edu.cn/admetsar2/ (accessed on 15 July 2024)) [26].

### 2.4. Molecular Docking

Molecular docking is a widely used technique to predict the binding affinity and interaction modes between peptides and target proteins at the molecular level [27]. In this study, to investigate the binding interactions between the screened peptides and ACE, molecular docking was performed using AutoDock Vina (version 1.1.2). The crystal structure of human ACE (PDB ID: 1O86) was obtained from the RCSB Protein Data Bank (https://www.rcsb.org (accessed on 29 July 2024)). Prior to docking, all water molecules and original ligands were removed to prepare the receptor structure. The peptide structures were initially constructed using PyMol (version 3.1.0) and saved in the mol2 format, followed by hydrogen addition and water removal using AutoDockTools (version 4.2.6). The docking grid was set with a center at x = 40.4977, y = 34.8829, and z = 44.3814 based on the coordinates of the co-crystallized ligand, and a box size of 30 × 30 × 30 Å to fully cover the classical ACE active pocket, including the S1, S2, and S′ subsites. The peptide–ACE complexes with the lowest binding energies were subsequently visualized and analyzed using Discovery Studio Visualizer (v17.2.0) to identify key binding residues and interaction types.

### 2.5. Peptide Synthesis

The selected peptides were synthesized by HEFEI KS-V Peptide Biotechnology Co., Ltd. (Hefei, China) using solid-phase peptide synthesis. All peptides were purified to a minimum purity of 98%, and their identities and purities were further verified by high-performance liquid chromatography (HPLC) coupled with mass spectrometry (MS) prior to ACE-inhibitory activity evaluation.

### 2.6. Determination of ACE-Inhibitory Activity

The ACE-inhibitory activity of the peptides was measured according to a previously reported method [28]. Briefly, 50 μL of the peptide solution, prepared in borate buffer (pH 8.3) containing 0.3 mol/L NaCl, was mixed with 100 μL of HHL (5.82 mmol/L) prepared in the same buffer. After incubation at 37 °C for 5 min, the reaction was initiated by adding 15 μL of ACE solution (0.1 U/mL), also prepared in the borate buffer described above. The mixture was then incubated at 37 °C for 30 min and terminated by adding 150 μL of 1 mol/L HCl. For the blank control, the same procedure was performed using 50 μL of the borate buffer instead of the peptide solution.

The released hippuric acid (HA) was quantified using an Elite P3200 high-performance liquid chromatography (HPLC) system (Dalian Elite Analytical Instruments Co., Ltd., Dalian, China) equipped with a C18 reversed-phase column (Waters, Milford, MA, USA; 150 mm × 4.6 mm, 5 μm) at a detection wavelength of 228 nm. The mobile phase consisted of solvent A (water containing 0.05% trifluoroacetic acid) and solvent B (acetonitrile) in a ratio of 75:25 (*v*/*v*). Elution was performed under isocratic conditions at a flow rate of 0.5 mL/min for 25 min. Prior to injection, all samples were filtered through a 0.22 μm membrane.

The ACE inhibition rate (%) was calculated using the following equation:

(1) $$\mathrm{ACE}\;\mathrm{Inhibitory}\;\mathrm{rate}\left(\%\right)=\frac{A_{blank}-A_{sample}}{A_{blank}}\times 100$$

where A_blank_ is the peak area of HA in the blank control test, and A_sample_ is the peak area of HA in the sample test. Captopril was used as the positive control in this assay.

### 2.7. ACE Inhibition Mode Analysis

ACE inhibition kinetics of the peptide PYPDWSFAK was evaluated using Lineweaver–Burk plots. The peptide was tested at concentrations of 0, 72, and 225 μmol/L, and mixed with HHL substrate solutions at final concentrations of 0.5, 1, 2, and 5 mmol/L. The amount of HA produced in each assay was quantified by HPLC as described above. Lineweaver–Burk plots (1/V vs. 1/[S]) were used to determine kinetic parameters, including the maximum reaction velocity (V_max_) and the Michaelis constant (K_m_). The changes in these parameters were used to elucidate the inhibition pattern of the peptide on ACE.

### 2.8. Cell Viability

*Cell culture*. EA.hy926 endothelial cells were cultured in high-glucose Dulbecco’s Modified Eagle Medium (DMEM) supplemented with 10% fetal bovine serum (FBS) and 1% penicillin–streptomycin. Cells were maintained in a humidified incubator at 37 °C with 5% CO_2_. When cell confluence reached approximately 70–80%, subculturing was performed for subsequent experiments.

*Cytotoxicity assay*. The cytotoxicity of the peptides was evaluated using the MTT assay [29]. Briefly, EA.hy926 cells in the logarithmic growth phase were seeded into 96-well plates at a density of 5 × 10^4^ cells/mL (200 μL per well) and incubated at 37 °C with 5% CO_2_ for 24 h. After removing the medium, cells were treated with 200 μL of peptide or captopril solutions at various concentrations (0.0625, 0.125, 0.25, 0.5, and 1.0 mg/mL) prepared in complete medium, and incubated for an additional 24 h. Subsequently, the medium was discarded, and 20 μL of MTT solution (5 mg/mL) together with 80 μL of serum-free DMEM was added to each well. After incubation at 37 °C for 4 h, the supernatant was carefully removed, and 150 μL of DMSO was added to dissolve the formazan crystals. The plate was shaken for 10 min, and the absorbance was measured at 490 nm using a microplate reader. Cell viability was calculated using the following equation:

(2) $$\mathrm{Cell}\;\mathrm{viability}\left(\%\right)=\frac{A_{sample}-A_{blank}}{A_{control}-A_{blank}}\times 100$$

where A_sample_ represents the absorbance of cells treated with the peptide or captopril, A_control_ represents the absorbance of untreated cells, and A_blank_ represents the absorbance of wells containing medium without cells.

### 2.9. Wound Healing Assay

The effect of peptides on endothelial cell migration was evaluated using a wound healing assay [30]. Briefly, EA.hy926 cells were seeded into 6-well plates and cultured at 37 °C in a humidified atmosphere containing 5% CO_2_. When the cells reached full confluence, they were treated with 1 μmol/L Ang II for 12 h to induce endothelial dysfunction. Subsequently, a straight scratch was created across the cell monolayer using a sterile 200 μL pipette tip. Detached cells were gently removed by rinsing with PBS, leaving a clear cell-free wound area. To minimize proliferation interference, cells were then incubated in high-glucose DMEM containing 1% FBS, supplemented with different concentrations of the peptides or captopril. Images of the same wound area were captured at 0 h, 6 h, and 24 h using a fluorescence microscope (Axio Observer, Zeiss, Jena, Germany), and the wound area was quantified using ImageJ software (version 1.54g).

### 2.10. Determination of NO and ET-1 Levels

To evaluate the effect of the peptide on endothelial function, NO and ET-1 levels were measured in this study. Briefly, EA.hy926 cells were seeded in 6-well plates at a density of 4 × 10^5^ cells/well and allowed to adhere overnight. After removing the medium, cells were incubated with 1 μmol/L Ang II for 12 h to induce endothelial dysfunction. Subsequently, the peptide solutions at different concentrations (0.1, 0.2, and 0.4 mg/mL) or the captopril solutions were added, and the cells were incubated for an additional 24 h. For the blank control group, the cells received no treatment. After incubation, the culture supernatants were collected and centrifuged to remove debris. Both NO and ET-1 contents in the supernatants were quantified using commercial kits according to the manufacturer’s protocols. NO content was determined using the Griess reaction (OD = 550 nm), and ET-1 levels were measured via enzyme-linked immunosorbent assay (ELISA) (OD = 450 nm). Absorbance values were recorded at the corresponding wavelengths using a microplate reader (Spark 10M, Tecan, Männedorf, Switzerland).

### 2.11. Determination of SOD and GSH-Px Activities

The activities of SOD and GSH-Px were assessed as follows. EA.hy926 cells were seeded in 6-well plates at 4 × 10^5^ cells per well and cultured overnight. After attachment, the cells were serum-starved in high-glucose DMEM containing 1% FBS for 12 h, and then treated for 24 h with peptide solutions at the indicated concentrations or with captopril. Oxidative stress was subsequently induced by exposing the cells to 500 µmol/L hydrogen peroxide (H_2_O_2_) for 12 h. After treatment, the cells were washed with ice-cold PBS, lysed on ice, and centrifuged to collect the supernatants. SOD and GSH-Px activities were measured spectrophotometrically at 550 nm and 412 nm, respectively, using a microplate reader. Enzyme activities were calculated, normalized to total protein content, and expressed as U/mg protein.

### 2.12. Statistical Analysis

All data are presented as mean ± standard deviation (SD) from three independent experiments (*n* = 3). Statistical analyses were performed in GraphPad Prism 10 (GraphPad Software, San Diego, CA, USA). Group differences were evaluated by one-way analysis of variance (ANOVA) followed by Tukey’s post hoc test. A *p*-value < 0.05 was considered statistically significant.

## 3. Results and Discussion

### 3.1. In Silico Hydrolysis of RJPs

**Figure 1:**
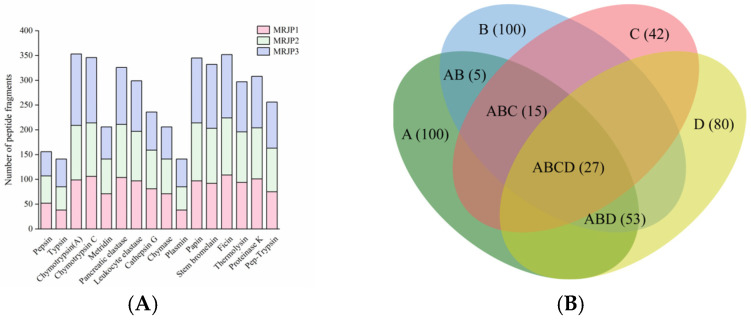
*In silico* hydrolysis of RJPs and virtual screening of RJP-derived peptides. (**A**) *In silico* hydrolysis of MRJP1, MRJP2, and MRJP3 using 15 individual proteases and 1 combined enzymatic system (pepsin + trypsin), showing the number of peptide sequences produced by each protease. (**B**) Venn diagram depicting the distribution of RJP-derived peptides based on predicted bioactivity and properties, in which the capital letters represent: A: PeptideRanker score > 0.8; B: non-toxicity; C: good water solubility; D: favorable HIA. Numbers in parentheses indicate the count of peptides in each category.

To fully generate a peptide sequence pool for further screening, MRJP1, MRJP2, and MRJP3 were subjected to *in silico* digestion using 15 different proteases and one combined enzymatic system (pepsin + trypsin). A total of 4300 peptide sequences were produced, with MRJP1, MRJP2, and MRJP3 yielding 1325, 1428, and 1547 peptides, respectively. Overall, plant proteases, including papain, stem bromelain, and ficin, generated a higher number of peptide sequences from MRJPs (345, 332, and 352 fragments, respectively; Figure 1A). This is likely because plant proteases possess broad substrate specificity, enabling cleavage at multiple sites, particularly at hydrophobic and aromatic residues that frequently occur in bioactive peptide motifs [31]. Similarly, microbial proteases generated slightly fewer peptides than plant proteases, with thermolysin and proteinase K producing 297 and 308 fragments, respectively. In contrast, animal proteases produced fewer peptide sequences overall, with an average yield of approximately 241 sequences per protease. Among these, chymotrypsin, which preferentially cleaves at aromatic residues, generated a substantial number of fragments (353 and 346 peptides for chymotrypsin A and chymotrypsin C, respectively), whereas enzymes with more restricted specificity, such as pepsin (156 sequences) and trypsin (141 sequences), produced markedly fewer fragments. Among the 4300 peptides generated, a considerable number were duplicated across different protease digestions; after removing redundant sequences, a total of 1411 unique peptides were retained for subsequent analyses.

### 3.2. Virtual Screening of the RJP-Derived Peptides

For the 1411 unique peptides derived from the *in silico* digests of RJPs, the potential bioactivity was initially assessed using PeptideRanker, an *in silico* tool that employs an N-to-1 neural network to assign a 0–1 probability score for peptide bioactivity. Because higher scores generally correlate with stronger biological effects, we applied a threshold of 0.8 to focus on peptides with a high likelihood of bioactivity. This filtering yielded 100 peptides, which were then subjected to further property analyses (Table S1).

To efficiently screen potential ACE-inhibitory peptides, key factors such as toxicity, water solubility, and HIA were prioritized, as these properties collectively influence peptide safety, release, absorption, and overall bioavailability. Among the 100 peptide candidates, all were predicted to be non-toxic, 43 peptides exhibited favorable water solubility, 80 peptides showed good HIA, and 27 peptides possessed all three favorable characteristics and were retained as optimal candidates (Figure 1B).

Analysis of the 27 selected peptides showed that their molecular weights ranged from approximately 200 to 1200 Da, with 85% below 1 kDa (Table 1). Low-molecular-weight peptides are frequently reported to exhibit stronger ACE-inhibitory activity, as their small size facilitates access to the narrow catalytic pocket of ACE and promotes effective binding [32]. The pI values of these peptides ranged from 3.8 to 10.11 (Table 1), and notably, 55% were basic (pI > 9) owing to the presence of Lys (K) and Arg (R) residues, which are frequently enriched, particularly at the C-terminus, in potent ACE inhibitors [8], possibly through electrostatic interactions with residues at allosteric or active sites of ACE.

Sequence composition analysis revealed that more than 85% of the peptides contained aromatic amino acids such as Phe (F), Tyr (Y), and Trp (W), which are often associated with enhanced ACE binding and stronger inhibitory activity. Notably, several peptides identified in this study, such as PYPDWSFAK, RPYPDWSF, QWHDKIF, FDVDRW, FRIM, and FRIL, carry multiple hydrophobic and aromatic residues, further supporting their strong ACE-inhibitory potential.

ADMET analysis indicated that all 27 selected peptides exhibited favorable human intestinal absorption, suggesting strong potential for oral bioavailability. In addition, ten peptides (FR, WR, DW, KF, DRW, QWR, FK, MR, RM, and EWKF) were predicted to cross the blood–brain barrier (Table 1), all of which were di-, tri- or tetra-peptides, consistent with the general observation that smaller peptides display greater membrane permeability. Predicted interactions with CYP2D6 showed that none of the peptides acted as substrates or inhibitors (Table 1), indicating a low risk of metabolic interference. Collectively, these physicochemical characteristics and ADMET profiles support the suitability of these peptides as promising lead compounds for ACE-inhibitor development.

### 3.3. Binding Affinity and in Vitro ACE-Inhibitory Activity

In this study, molecular docking was performed to evaluate the binding affinities of the 27 selected peptides and the positive control captopril with ACE, allowing a comparative assessment of their potential inhibitory effects. The binding affinity of captopril toward ACE was calculated as −5.6 kcal/mol (Table 2), which is consistent with the value reported by Han et al. (−5.54 kcal/mol) [33], thereby confirming the reliability of the docking protocol used in this study. All 27 peptides exhibited binding affinities lower than that of captopril, suggesting that they could form more stable peptide–ACE complexes. The peptide binding affinities ranged from −10.2 to −6.1 kcal/mol (Table 2), with nearly two-thirds (63.0%) displaying values below −8.0 kcal/mol, indicating strong ACE-inhibitory potential. This trend aligns with previous findings in which 37 peptides identified from the gel chromatography fraction of the alkaline protease hydrolysate of *Torreya grandis* demonstrated comparable docking affinities (−7.0 to −10.0 kcal/mol), further supporting the robustness of the present docking results [34].

**Figure 2:**
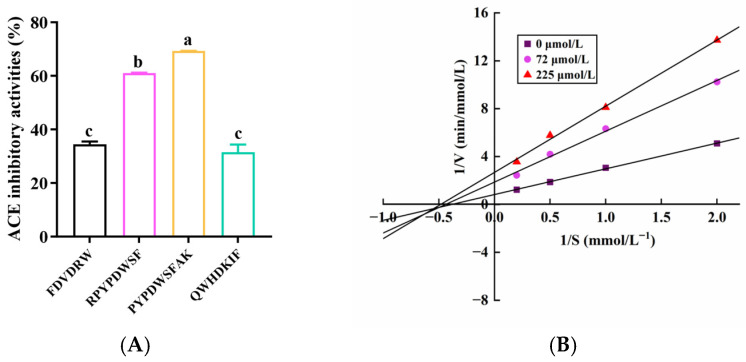
ACE-inhibitory activity and kinetic characterization of the selected peptides. (**A**) ACE-inhibitory activity of the four peptides exhibiting the lowest binding affinities in molecular docking, each tested at 0.5 mg/mL. Superscript letters on the bars indicate significant differences between groups (*p* < 0.05). (**B**) Lineweaver–Burk plot illustrating the inhibition kinetics of PYPDWSFAK against ACE for the determination of the inhibition mode and kinetic parameters.

To further validate the ACE-inhibitory activities of the peptides selected based on *in silico* screening, the top four peptides with the lowest binding affinities (<−10.0 kcal/mol) were synthesized and subjected to an *in vitro* ACE-inhibitory assay. The results showed that the peptides PYPDWSFAK and RPYPDWSF exhibited significantly higher ACE-inhibitory activity *in vitro* than FDVDRW and QWHDKIF, despite the latter displaying comparable or slightly stronger predicted binding affinities in molecular docking. Furthermore, IC_50_ values were determined for the two most active peptides, with PYPDWSFAK and RPYPDWSF showing IC_50_ values of 110 ± 1.02 μmol/L and 204 ± 0.61 μmol/L, respectively, indicating their strong potential as ACE inhibitors (Figure 2A). Notably, their activities were markedly superior to several previously reported food-derived peptides. For instance, LPK (IC_50_ = 6.54 mmol/L) and PRP (IC_50_ = 0.42 mmol/L) isolated from *Sipunculus nudus* L. [35], as well as SEGPK (IC_50_ = 1.21 mmol/L), FDGPY (IC_50_ = 1.57 mmol/L), and SPGPW (IC_50_ = 1.31 mmol/L) identified from *Lophius litulon* [36], all exhibited substantially weaker ACE-inhibitory activity. These comparisons underscore the superior potency of PYPDWSFAK and RPYPDWSF and reinforce their promise as natural antihypertensive candidates.

Among the two peptides, PYPDWSFAK exhibited higher ACE-inhibitory activity, which may be closely related to its amino acid composition. Studies have shown that peptides enriched in hydrophobic amino acids, especially those containing aromatic side chains, tend to display stronger ACE-inhibitory potential [37]. In addition, proline is considered a key residue that enhances peptide stability and biological activity [38]. PYPDWSFAK contains two proline residues, which may further contribute to its potency. Moreover, its balanced distribution of hydrophobic and hydrophilic residues confers a favorable structural feature reported to facilitate interaction with the ACE catalytic pocket and enhance inhibitory efficiency [39]. Taken together, the presence of multiple proline residues, abundant hydrophobic/aromatic amino acids, and a favorable amphiphilic profile may underlie the strong ACE-inhibitory activity observed for this peptide. Therefore, PYPDWSFAK was selected for further investigation to elucidate its inhibitory mechanism.

### 3.4. ACE Inhibition Pattern of PYPDWSFAK

Given that PYPDWSFAK exhibited the strongest ACE-inhibitory activity, its inhibition mechanism was further investigated using enzyme kinetic analysis. As shown in the Lineweaver–Burk plots (Figure 2B), increasing concentrations of PYPDWSFAK led to a marked decrease in the V_max_, demonstrating that the peptide may restrict substrate access to the active site of ACE (Table 3). Meanwhile, K_m_ showed a slight increase, suggesting a lowered apparent affinity between ACE and its substrate in the presence of the peptide. The concurrent decrease in V_max_ and increase in K_m_ indicates that PYPDWSFAK acts as a mixed-type ACE inhibitor, capable of interacting with both the catalytic site and an allosteric site, thereby impairing substrate binding and catalytic turnover simultaneously. Similar mixed-type ACE inhibition mechanisms have been reported for other food-derived peptides, including KWEKPF from Chinese Rushan cheese by-products [40], LTGCP from tuna (*Thunnus thynnus*) muscle [41] and LEPWR from Pacific saury [42]. These findings collectively support the potential of PYPDWSFAK to inhibit ACE through multifaceted inhibitory mechanisms.

### 3.5. Docking Conformation of PYPDWSFAK-ACE Complex

**Figure 3:**
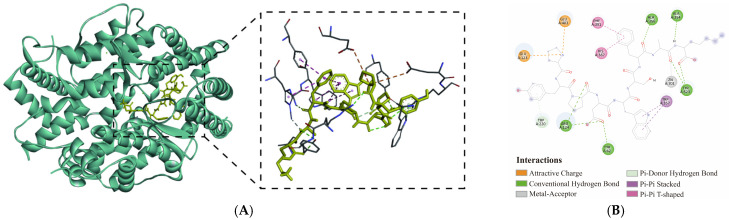
Docking conformation of the PYPDWSFAK-ACE complex. (**A**) 3D binding conformation of the PYPDWSFAK-ACE complex. (**B**) 2D interaction diagram illustrating the key hydrogen bonds, hydrophobic and electrostatic interactions between PYPDWSFAK and ACE.

To further elucidate the inhibitory mechanism of PYPDWSFAK against ACE, the docking conformation of the PYPDWSFAK-ACE complex with the lowest binding affinity was visualized (Figure 3A). Generally, ACE contains a well-characterized active pocket composed of three subsites (S1, S2, and S′1 subsite) that are critical for substrate binding and catalysis. The S1 subsite includes residues Ala354, Glu384, and Tyr523; the S2 subsite comprises Gln281, His353, Lys511, His513, and Tyr520; and the S′1 subsite contains only Glu162 [6]. In addition, ACE features a catalytic zinc ion (Zn^2+^) coordinated by His383, His387, and Glu411, forming the conserved metal-binding core essential for enzymatic activity [43].

Based on docking analysis, PYPDWSFAK formed a stable complex with ACE, primarily through hydrogen bonding, supplemented by electrostatic and hydrophobic interactions (Figure 3B). Eight hydrogen bonds were identified, including 3 hydrogen bonds with key S1 pocket residues (Ala354, Tyr523) and 5 hydrogen bonds with additional residues outside the catalytic pocket (Arg124, Tyr62, Trp220, Ala356). Beyond hydrogen bonding, two electrostatic interactions (Glu123, Glu403) and four hydrophobic contacts (Trp357, His387, Phe390) further stabilized the complex (Table S2). Notably, PYPDWSFAK also coordinated directly with the catalytic Zn701, a feature strongly associated with potent ACE inhibition [14]. Classical inhibitors such as captopril similarly coordinate with Zn^2+^ within the HEXXH motif, underscoring the functional significance of this interaction [44].

Similar binding patterns have been reported previously. For example, SP and VDRYF from skipjack tuna muscle formed hydrogen bonds with Ala354 and Tyr523 within S1 pocket, respectively [45]. SEGPK and FDGPY from monkfish swim bladder interacted with Ala354 and Tyr523 via hydrogen bonding and hydrophobic interactions, while SPGPW formed a hydrogen bond with the catalytic residue His387 [36]. Likewise, ITAPHW and SLPNYHPSPR from fermented black sesame seed displayed strong ACE inhibition due to direct Zn^2+^ coordination [46]. These comparisons support the interaction profile observed for PYPDWSFAK in this study.

Taken together, the interaction pattern suggests that PYPDWSFAK inhibits ACE by engaging both the catalytic pocket and an adjacent allosteric region, consistent with the mixed-type inhibition pattern observed in kinetic studies. Moreover, its strong *in vitro* inhibitory activity appears to arise from the combination of direct Zn^2+^ coordination and robust interactions with key S1 pocket residues, which together enhance the stability of the peptide–ACE complex.

### 3.6. Cytotoxicity of PYPDWSFAK on EA.hy926 Cells

Endothelial dysfunction activates multiple signaling pathways that drive abnormal vascular cell proliferation and remodeling, ultimately contributing to the development of hypertension [47]. EA.hy926 cells, due to their endothelial characteristics, have been widely used as an *in vitro* model to investigate endothelial dysfunction and hypertension-related mechanisms [48,49].

**Figure 4:**
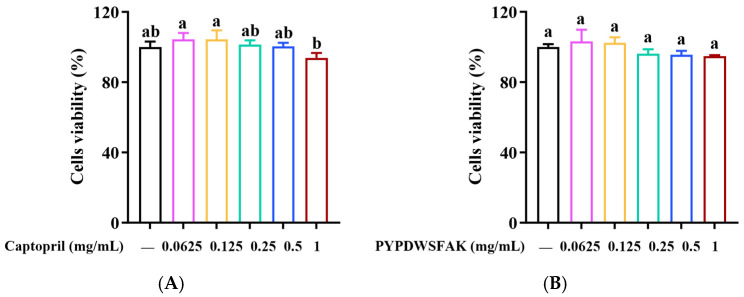
Effects of PYPDWSFAK (**A**) and captopril (**B**) on the viability of EA.hy926 cells. Different lowercase letters above the bars indicate significant differences within the same treatment group (*p* < 0.05).

To evaluate the cytocompatibility of the peptide PYPDWSFAK and the positive control captopril, an MTT assay was performed after 24 h of treatment with each compound at concentrations ranging from 0.0625 to 1 mg/mL. As shown in Figure 4A,B, PYPDWSFAK-treated cells exhibited viabilities ranging from 94.84% to 103.08%, while captopril-treated cells showed viabilities between 93.87% and 104.41%, with no obvious variation detected within either treatment group. According to ISO 10993-5 guidelines, cell viability above 70% is considered non-cytotoxic [50]. Therefore, the peptide PYPDWSFAK can be regarded as non-cytotoxic toward EA.hy926 cells under the tested conditions, supporting the safety profile of PYPDWSFAK and its potential applicability in antihypertensive therapy development.

### 3.7. Effect of PYPDWSFAK on EA.hy926 Cell Migration

Abnormal endothelial cell migration is implicated in vascular remodeling and neointimal formation, both of which contribute to the progression of hypertension and its associated vascular complications [51]. Therefore, the inhibitory effect of PYPDWSFAK on cell migration was evaluated using a wound healing assay in EA.hy926 cells.

**Figure 5:**
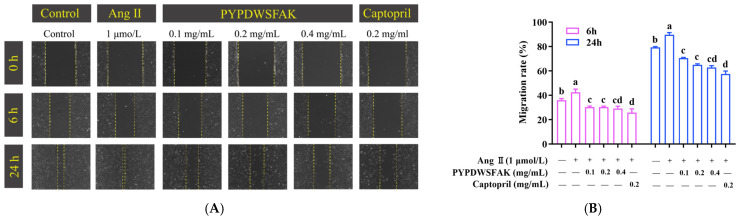
Effects of PYPDWSFAK on Ang II-induced endothelial cell migration. (**A**) Representative wound-healing images of EA.hy926 cells treated with Ang II alone or co-treated with PYPDWSFAK (0.1, 0.2, 0.4 mg/mL) or captopril (0.2 mg/mL, positive control) at 0, 6, and 24 h. (**B**) Migration rates at 6 h and 24 h under each condition, quantified from wound area measurements in ImageJ. Different lowercase letters denote significant differences between groups (*p* < 0.05).

As shown in Figure 5, Ang II stimulation significantly accelerated wound closure compared to the control group. At 6 h and 24 h post-scratch, the migration rates in the Ang II group reached 42.44 ± 2.12% and 89.68 ± 1.47%, respectively, whereas the control group showed rates of 35.95 ± 1.08% and 79.33 ± 0.48%. This indicates that Ang II promotes abnormal endothelial cell migration, which may contribute to vascular hyperplasia and remodeling during hypertension. At 6 h, PYPDWSFAK exhibited a clear inhibitory effect on Ang II-induced migration. All three concentrations produced highly similar suppression, with migration rates of 30.32 ± 0.64%, 30.29 ± 0.59%, and 29.03 ± 1.54%, respectively. These values were substantially lower than those of the Ang II group and were comparable to the captopril-treated group (25.77 ± 2.49%). At 24 h, the inhibitory pattern became concentration-dependent. The migration rates for the 0.1, 0.2, and 0.4 mg/mL PYPDWSFAK groups were 70.57 ± 0.38%, 64.99 ± 0.63%, and 62.87 ± 1.12%, respectively, all significantly lower than that of the Ang II group (89.68 ± 1.47%), demonstrating a sustained anti-migratory effect. Notably, the peptide at the highest concentration (0.4 mg/mL) produced the strongest inhibition and exhibited an activity level comparable to captopril (57.52 ± 1.92%). This trend is consistent with previous findings showing that food-derived pep-tides can alleviate Ang II-induced endothelial injury. For instance, the soybean protein-derived peptide SPIH restored endothelial wound healing from 129% in the Ang II group to 92% after 48 h of treatment [52]. Collectively, these findings demonstrate that the peptide PYPDWSFAK effectively counteracts Ang II-induced endothelial cell migration and suggest that it may help mitigate vascular remodeling under hypertensive conditions.

### 3.8. Effect of PYPDWSFAK on NO and ET-1 Levels in Ang II-Stimulated EA.hy926 Cells

Endothelial dysfunction, characterized by reduced NO bioavailability and elevated ET-1 production, is a central feature in the pathogenesis of hypertension [53]. In this study, Ang II was applied to EA.hy926 cells to mimic endothelial dysfunction, as it is known to reduce NO levels and enhance ET-1 expression [54].

**Figure 6:**
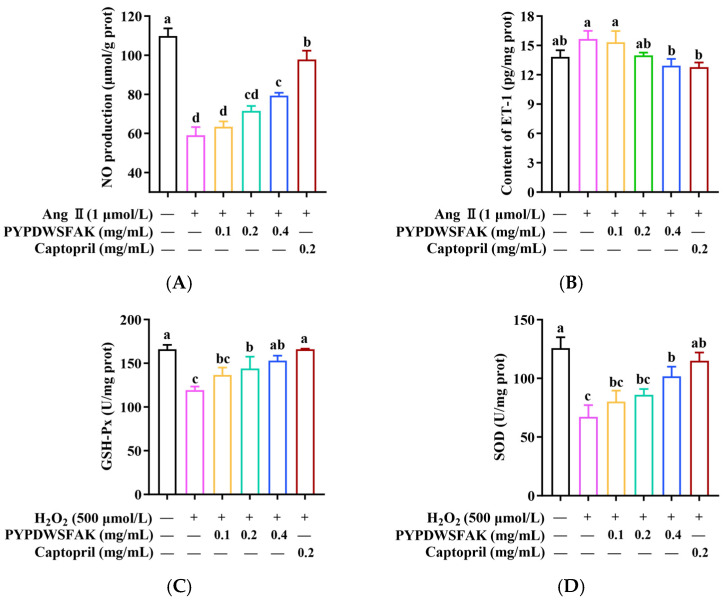
Effects of PYPDWSFAK on NO (**A**) and ET-1 (**B**) levels in Ang II-induced EA.hy926 cells, and on GSH-Px (**C**) and SOD (**D**) activities in H_2_O_2_-damaged EA.hy926 cells. Different lowercase letters indicate significant differences between groups (*p* < 0.05).

As shown in Figure 6A, Ang II treatment significantly suppressed NO production, reducing its level to 58.95 ± 4.31 μmol/g prot compared to the control group (109.8 ± 3.93 μmol/g prot) (*p* < 0.05). Treatment with PYPDWSFAK led to a concentration-dependent increase in NO, with the highest level observed at 0.4 mg/mL (79.36 ± 1.48 μmol/g prot). Although this value was slightly lower than that achieved with captopril (97.86 ± 4.47 μmol/g prot), the peptide still showed a clear capacity to restore NO levels under Ang II-induced stress. Meanwhile, Ang II stimulation markedly elevated ET-1 secretion, reaching 15.65 ± 0.83 pg/mg prot, which was significantly higher than that of the control group (13.82 ± 0.69 pg/mg prot). PYPDWSFAK treatment attenuated this increase in a dose-dependent manner (Figure 6B). At 0.4 mg/mL, ET-1 content decreased to 12.93 ± 0.69 pg/mg prot, a level closely comparable to that of the captopril group (12.70 ± 0.48 pg/mg prot). These results indicate that PYPDWSFAK is capable of restoring the NO/ET-1 balance disrupted by Ang II, thereby alleviating endothelial dysfunction. Similar regulatory effects have been reported for other food-derived peptides. For example, the soybean protein–derived peptide SPIH increased NO content in Ang II–treated endothelial cells from 31.4 ± 0.7 to 43.7 ± 0.1 μmol/L, corresponding to a 39.3% increase [52], a magnitude comparable to the 34.5% improvement observed for PYPDWSFAK in this study. Taken together, these findings suggest that PYPDWSFAK exerts a protective effect on endothelial cells under hypertensive conditions by simultaneously enhancing NO production, possibly through eNOS activation as indicated by transcriptomic analysis, and suppressing ET-1 secretion.

### 3.9. Effect of PYPDWSFAK on GSH-Px and SOD Activities in H_2_O_2_-Damaged EA.hy926 Cells

Oxidative stress has been increasingly recognized as a critical factor in the pathogenesis of vascular injury and the development of hypertension [55]. Among various *in vitro* oxidative stress models, H_2_O_2_ is commonly used as an inducer owing to its availability, stability, and capacity to mimic physiological oxidative damage [56]. SOD and GSH-Px serve as essential enzymatic antioxidants, and their activities are widely regarded as reliable indicators of cellular antioxidant defense mechanisms [57,58].

In this study, oxidative stress was induced in EA.hy926 cells using 500 μmol/L H_2_O_2_, and the protective effects of PYPDWSFAK were evaluated by measuring SOD and GSH-Px activities. As shown in Figure 6C,D, H_2_O_2_ treatment significantly reduced GSH-Px activity to 119.49 ± 3.94 U/mg prot compared to the control (165.92 ± 5.21 U/mg prot), and SOD activity to 67.47 ± 9.93 U/mg prot versus 126.15 ± 9.22 U/mg prot in the control. PYPDWSFAK treatment restored enzyme activities in a concentration-dependent manner, reaching 153.10 ± 5.70 U/mg prot for GSH-Px and 101.63 ± 8.25 U/mg prot for SOD at 0.4 mg/mL, corresponding to a 28.1% and 50.6% recovery, respectively. Compared with captopril (38.8% improvement for GSH-Px, and 70.9% improvement for SOD), PYPDWSFAK showed slightly lower recovery. These observations are in line with previously reported antioxidant peptides, such as GEYGFE, PSVSLT, and GIELFPGLP from *Acipenser baerii*, which similarly enhanced SOD (36.5–53.2%) and GSH-Px (12.1–33.6%) activities under oxidative challenge [59]. Overall, these findings suggest that PYPDWSFAK confers antioxidative protection in endothelial cells by enhancing the activities of antioxidant enzymes such as SOD and GSH-Px, thereby contributing to the maintenance of endothelial function, possibly via the Nrf2 pathway, as reported previously [60].

## 4. Conclusions

This study successfully identified novel ACE-inhibitory peptides from RJPs through an integrated strategy combining *in silico* digestion, virtual screening, molecular docking, and *in vitro* validation. Among the screened candidates, PYPDWSFAK exhibited the strongest inhibitory activity, characterized by mixed-type inhibition and the formation of multiple hydrogen bonds with key residues in the ACE active pocket, along with direct coordination to the catalytic Zn^2+^ ion. In cellular assays, PYPDWSFAK demonstrated excellent safety, effectively suppressed Ang II–induced endothelial cell migration, restored NO and ET-1 homeostasis, and enhanced antioxidant enzyme activities under oxidative stress. These findings indicate that RJPs represent a valuable source of bioactive peptides and identify PYPDWSFAK as a particularly promising candidate for development as a natural antihypertensive ingredient, although further *in vivo* studies are still needed. Moreover, the integrated workflow established in this study provides an efficient and rational approach for discovering functional peptides from diverse food protein sources.

## Figures and Tables

**Table 1:** Physicochemical properties, toxicity, and ADMET profiles of the 27 selected peptides derived from the *in silico* digests of MRJP1, MRJP2, and MRJP3.

No.	Sequence	Molecular Weight (g/mol)	Scores ^1^	Toxicity ^2^	Water Solubility	pI	Net Charge at pH 7.0	HIA ^3^	BBB ^4^	CYP450 2D6 Interaction ^5^
1	FR	321.39	0.99	Non-toxic	Good	10.11	1	+	+	-
2	WR	360.43	0.98	Non-toxic	Good	10.11	1	+	+	-
3	RWL	473.61	0.95	Non-toxic	Good	10.11	1	+	-	-
4	PDW	416.46	0.95	Non-toxic	Good	3.8	−1	+	-	-
5	DW	319.33	0.93	Non-toxic	Good	3.8	−1	+	+	-
6	KPYPDWSF	1039.25	0.93	Non-toxic	Good	6.18	0	+	-	-
7	FRIM	565.78	0.92	Non-toxic	Good	10.11	1	+	-	-
8	RPYPDWSF	1067.26	0.91	Non-toxic	Good	6.19	0	+	-	-
9	KF	293.38	0.91	Non-toxic	Good	9.11	1	+	+	-
10	PYPDWSFAK	1110.34	0.90	Non-toxic	Good	6.18	0	+	-	-
11	DRW	475.53	0.89	Non-toxic	Good	6.19	0	+	+	-
12	FHR	458.55	0.88	Non-toxic	Good	10.11	1.5	+	-	-
13	QWR	488.58	0.87	Non-toxic	Good	10.11	1	+	+	-
14	FDL	393.47	0.87	Non-toxic	Good	3.8	−1	+	-	-
15	FHRL	571.73	0.87	Non-toxic	Good	10.11	1.5	+	-	-
16	FK	293.38	0.86	Non-toxic	Good	9.11	1	+	+	-
17	MR	305.41	0.85	Non-toxic	Good	10.11	1	+	+	-
18	QWHDKIF	973.2	0.85	Non-toxic	Good	7.09	0.5	+	-	-
19	RM	305.41	0.85	Non-toxic	Good	10.11	1	+	+	-
20	FRIL	547.75	0.85	Non-toxic	Good	10.11	1	+	-	-
21	MTRW	592.76	0.84	Non-toxic	Good	10.11	1	+	-	-
22	KWL	445.6	0.84	Non-toxic	Good	9.11	1	+	-	-
23	FDVDRW	836.97	0.83	Non-toxic	Good	4.21	−1	+	-	-
24	KNYPF	667.82	0.83	Non-toxic	Good	8.94	1	+	-	-
25	FDYDPKFT	1032.21	0.83	Non-toxic	Good	4.21	−1	+	-	-
26	RP	271.33	0.82	Non-toxic	Good	10.11	1	+	-	-
27	EWKF	608.74	0.80	Non-toxic	Good	6.35	0	+	+	-

^1^ Scores, PeptideRanker Scores, predicted using the PeptideRanker program; higher scores indicate a greater likelihood of biological activity. ^2^ Toxicity, predicted using the ToxinPred program. ^3^ HIA, human intestinal absorption; “-” in the column stands for “no HIA”, and + stands for “HIA”. ^4^ BBB, Blood–Brain Barrier; “-” denotes peptides unlikely to cross the Blood–Brain Barrier, while “+” denotes predicted BBB penetration. ^5^ CYP450 2D6 interaction, cytochrome P450 2D6 interaction; “-” represents peptides predicted not to inhibit CYP450 2D6, and “+” represents predicted inhibitors.

**Table 2:** Predicted binding affinities of the 27 selected peptides with ACE.

No.	Peptide Sequence	Binding Affinity (kcal/mol)	No.	Peptide Sequence	Binding Affinity (kcal/mol)
1	FDVDRW	−10.2	15	FRIM	−8.5
2	RPYPDWSF	−10.1	16	RWL	−8.4
3	PYPDWSFAK	−10	17	PDW	−8.2
4	QWHDKIF	−10	18	FDYDPKFT	−7.9
5	FHRL	−9.9	19	FR	−7.8
6	EWKF	−9.4	20	KWL	−7.7
7	QWR	−8.9	21	FDL	−7.7
8	FRIL	−8.9	22	FK	−7.4
9	FHR	−8.8	23	DW	−7.1
10	WR	−8.7	24	RP	−6.9
11	KPYPDWSF	−8.7	25	KF	−6.5
12	MTRW	−8.7	26	RM	−6.2
13	KNYPF	−8.7	27	MR	−6.1
14	DRW	−8.5	28	Captopril	−5.6

**Table 3:** Kinetic parameters V_max_ and K_m_ of the peptide PYPDWSFAK at various concentrations.

Parameter	Concentrations of the Peptide PYPDWSFAK		
	0 μmol/L	72 μmol/L	225 μmol/L
V_max_ (mmol/L/min)	1.21	0.54	0.37
K_m_ (mmol/L)	2.61	2.28	2.06

## Data Availability

The original contributions presented in this study are included in the article/Supplementary Materials. Further inquiries can be directed to the corresponding author.

## Supplementary Materials

The following supporting information can be downloaded at: https://www.mdpi.com/article/10.3390/foods15010084/s1, Table S1: Peptide sequences selected by PeptideRanker (score > 0.8) and their predicted toxicity, solubility, and HIA; Table S2: Residue-specific interactions between peptide PYPDWSFAK and ACE at the active subsites and potential allosteric subsites.
