# Supplementary material for: Novel ACE-Inhibitory Peptides from Royal Jelly Proteins: Comprehensive Screening, Mechanistic Insights, and Endothelial Protection
Source: Foods. 2025 Dec 26;15(1):84. doi: 10.3390/foods15010084 (PMC12785803; doi:10.3390/foods15010084)
Supplement: Supplementary file 1 [file foods-15-00084-s001.zip › foods-4022937-supplementary.pdf]

Table S1: Peptide sequences selected by PeptideRanker (score > 0.8) and their predicted toxicity, solubility, and HIA

Number	Sequence	PeptideRanker score	Toxicity	Water solubility	HIA
1	FM	0.996	Non-Toxin	Poor	+
2	FFG	0.996	Non-Toxin	Poor	+
3	PF	0.993	Non-Toxin	Poor	+
4	FG	0.993	Non-Toxin	Poor	+
5	GCW	0.993	Non-Toxin	Poor	+
6	WLLLVVCLG IACQDVTSAA VNHQR	0.991	Non-Toxin	Poor	+
7	FFGL	0.990	Non-Toxin	Poor	+
8	FL	0.990	Non-Toxin	Poor	+
9	WL	0.987	Non-Toxin	Poor	+
10	FR	0.986	Non-Toxin	Good	+
11	LW	0.985	Non-Toxin	Poor	+
12	FML	0.985	Non-Toxin	Poor	+
13	YF	0.982	Non-Toxin	Poor	+
14	WR	0.978	Non-Toxin	Good	+
15	AF	0.973	Non-Toxin	Poor	+
16	APF	0.967	Non-Toxin	Poor	+
17	CGM	0.959	Non-Toxin	Poor	-
18	FA	0.956	Non-Toxin	Poor	+
19	PDWSF	0.954	Non-Toxin	Poor	+
20	FN	0.951	Non-Toxin	Poor	-
21	HF	0.951	Non-Toxin	Poor	+
22	PYPDWSF	0.951	Non-Toxin	Poor	+
23	IF	0.949	Non-Toxin	Poor	+
24	SF	0.949	Non-Toxin	Poor	+
25	RWL	0.947	Non-Toxin	Good	+
26	QF	0.946	Non-Toxin	Poor	-
27	PDW	0.945	Non-Toxin	Good	+
28	MG	0.944	Non-Toxin	Poor	+
29	DF	0.942	Non-Toxin	Good	-
30	NF	0.941	Non-Toxin	Poor	-
31	PHF	0.938	Non-Toxin	Poor	+
32	PIF	0.935	Non-Toxin	Poor	+
33	QPYPDWSF	0.934	Non-Toxin	Poor	+
34	NYPF	0.933	Non-Toxin	Poor	+
35	DW	0.933	Non-Toxin	Good	+
36	CG	0.932	Non-Toxin	Poor	+
37	KPYPDWSF	0.927	Non-Toxin	Good	+
38	DFDFG	0.925	Non-Toxin	Good	-
39	FD	0.922	Non-Toxin	Good	-
40	FRIM	0.921	Non-Toxin	Good	+

Table S1 (Continued): Peptide sequences selected by PeptideRanker (score > 0.8) and their predicted toxicity, solubility, and HIA

Number	Sequence	PeptideRanker score	Toxicity	Water solubility	HIA
41	QFG	0.919	Non-Toxin	Poor	+
42	RPYPDWSF	0.912	Non-Toxin	Good	+
43	DFG	0.910	Non-Toxin	Good	-
44	KF	0.907	Non-Toxin	Good	+
45	SGVLFFGLVG DSALGCWNE HR	0.904	Non-Toxin	Poor	+
46	PYPDWSFAK	0.902	Non-Toxin	Good	+
47	ML	0.895	Non-Toxin	Poor	+
48	GGPL	0.893	Non-Toxin	Poor	+
49	DRW	0.889	Non-Toxin	Good	+
50	FMV	0.885	Non-Toxin	Poor	+
51	ICGM	0.883	Non-Toxin	Poor	+
52	TPF	0.882	Non-Toxin	Poor	+
53	GICGM	0.881	Non-Toxin	Poor	+
54	CL	0.880	Non-Toxin	Poor	+
55	FHR	0.879	Non-Toxin	Good	+
56	QWR	0.871	Non-Toxin	Good	+
57	CGR	0.871	Non-Toxin	Good	-
58	FDL	0.870	Non-Toxin	Good	+
59	FHRL	0.868	Non-Toxin	Good	+
60	FK	0.860	Non-Toxin	Good	+
61	CGMA	0.856	Non-Toxin	Poor	-
62	FGK	0.853	Non-Toxin	Good	-
63	NGVLFVGLV GNSAVGCWN EHQSLQR	0.851	Non-Toxin (0.70)	Poor	+
64	MR	0.849	Non-Toxin	Good	+
65	QWHDKIF	0.848	Non-Toxin	Good	+
66	RM	0.848	Non-Toxin	Good	+
67	FRIL	0.848	Non-Toxin	Good	+
68	FDY	0.847	Non-Toxin	Good	-
69	FDR	0.847	Non-Toxin	Good	-
70	QPMCSPKL	0.846	Non-Toxin	Good	-
71	WLFMVACLG IACQGAIVR	0.845	Non-Toxin	Poor	+
72	MY	0.843	Non-Toxin	Poor	+
73	MTRW	0.840	Non-Toxin	Good	+
74	GGL	0.839	Non-Toxin	Poor	+
75	KWL	0.837	Non-Toxin	Good	+
76	YM	0.837	Non-Toxin	Poor	+
77	DPKF	0.835	Non-Toxin	Good	-

Table S1 (Continued): Peptide sequences selected by PeptideRanker (score > 0.8) and their predicted toxicity, solubility, and HIA

Number	Sequence	PeptideRanker score	Toxicity	Water solubility	HIA
78	SPM	0.834	Non-Toxin	Poor	+
79	RCG	0.833	Non-Toxin	Good	-
80	FDRY	0.833	Non-Toxin	Good	-
81	KFFDY	0.831	Non-Toxin	Good	-
82	MTRWL	0.829	Non-Toxin	Poor	+
83	FDVDRW	0.827	Non-Toxin	Good	+
84	TF	0.827	Non-Toxin	Poor	+
85	KNYPF	0.826	Non-Toxin	Good	+
86	FDYDPKFT	0.825	Non-Toxin	Good	+
87	VGDGGPLLQ PYPDWSFAK	0.823	Non-Toxin	Poor	+
88	RP	0.822	Non-Toxin	Good	+
89	FT	0.822	Non-Toxin	Poor	+
90	SAF	0.821	Non-Toxin	Poor	+
91	AGM	0.820	Non-Toxin	Poor	+
92	SAVGCW	0.817	Non-Toxin	Poor	+
93	VF	0.815	Non-Toxin	Poor	+
94	GGR	0.811	Non-Toxin	Good	-
95	PL	0.811	Non-Toxin	Poor	+
96	QPM	0.810	Non-Toxin	Poor	+
97	GL	0.809	Non-Toxin	Poor	+
98	PYPDWS	0.806	Non-Toxin	Poor	+
99	EWKF	0.803	Non-Toxin	Good	+
100	GDGGPL	0.800	Non-Toxin	Good	-

Table S2: Residue-specific interactions between peptide PYPDWSFAK and ACE at the active subsites and potential allosteric subsites

Interaction types	Active subsite	Potential allosteric subsite
Hydrogen bonds	Lys^{9}-Ala354 (S1); Lys^{9}-Tyr523 (S1);	Pro^{1} (O)-Arg124; Tyr^{2}-Trp220; Asp^{4}-Tyr62; Asp^{4}-Arg124; Phe^{7} (O)-Ala356; Ala^{8} (O)-Tyr523;
Electrostatic interactions		Pro^{1}-Glu123; Pro^{1}-Glu403;
Hydrophobic interactions	Phe^{7}-His383 (Zn^{2+}-coordinating residue);	Phe^{7}-Phe391
Metal coordination	Ala^{8} (O)-Zn701;	

Note: Interaction pairs are reported as peptide residue–ACE residue (the residue before the dash belongs to the peptide PYPDWSFAK, and the residue after the dash belongs to ACE). Superscripts on the residues (e.g., Pro^{1}, Tyr^{2}, Asp^{4}, Phe^{7}, Ala^{8} and Lys^{9}) indicate the residue position in PYPDWSFAK. ACE subsites are given in parentheses after ACE residues (e.g., Ala354 (S1), Tyr523 (S1)). (O) denotes the backbone carbonyl oxygen of the peptide residue involved in the interaction.
